# Advances in Pharmaceutical Strategies Enhancing the Efficiencies of Oral Colon-Targeted Delivery Systems in Inflammatory Bowel Disease

**DOI:** 10.3390/molecules23071622

**Published:** 2018-07-04

**Authors:** Yilin Guo, Shiyu Zong, Yiqiong Pu, Benliang Xu, Tong Zhang, Bing Wang

**Affiliations:** 1 Experiment Center for Teaching and Learning, Shanghai University of Traditional Chinese Medicine, 1200 Cailun Road, Pudong New District, Shanghai 201203, China; gyl101010@126.com (Y.G.); zongsy114@163.com (S.Z.); puyiq@163.com (Y.P.); xbl2004000@126.com (B.X.); 2 School of Pharmacy, Shanghai University of Traditional Chinese Medicine, 1200 Cailun Road, Pudong New District, Shanghai 201203, China; 3 Shaanxi Academy of Traditional Chinese Medicine, Xi’an 710003, Shaanxi, China

**Keywords:** targeted drug-delivery strategies, treatment, inflammatory bowel disease

## Abstract

Inflammatory bowel disease (IBD) is a common disease characterized by chronic inflammation in gastrointestinal tracts, which is primarily treated by administering anti-inflammatory and immunosuppressive drugs that inhibit the burden of intestinal inflammation and improve disease-related symptoms. However, the established therapeutic strategy has limited therapeutic efficacy and adverse drug reactions. Therefore, new disease-targeting drug-delivery strategies to develop more effective treatments are urgent. This review provides an overview of the drug-targeting strategies that can be used to treat IBD, and our recent attempts on the colon-specific delivery system (Pae-SME-CSC) with a paeonol-loaded self-microemulsion (Pae-SMEDDS) are introduced.

## 1. Introduction

Inflammatory bowel disease (IBD) includes two major types of disease: Crohn’s disease (CD) and ulcerative colitis (UC) [1,2], which are chronic recurrent inflammatory diseases of the gastrointestinal tract involving the large intestine or colon [3]. UC and CD are considered different conditions; however, they share some common clinical features, such as cycles of relapse and remitting mucosal inflammation [4]. For UC, the inflammation is restricted to the colon and rectum continuously, with some cases even reaching the whole colon. Ulcerative colitis is one of the most common causes of colorectal cancer. Carcinogenesis is related to the time limit and extent of lesions of ulcerative colitis. The longer the disease course, the greater the chance of carcinogenesis in active cases with more extensive lesions. The incidence of ulcerated colorectal cancer is significantly higher than that of the general population. In the process of inflammatory hyperplasia, inflammatory or pseudopolyps are often formed and cancer occurs. However, it takes a long time and the cancerous rate of this colonic polyp is low. The occurrence of cancerous changes in the karyotype is more common in the undifferentiated type, with a higher degree of malignancy and a poorer prognosis [5]. CD affects any region of the GI tract, with the terminal ileum and the colon commonly affected, and the inflammation is generally noncontinuous [6,7]. The exact cause of IBD is uncertain, but some factors have been suggested, such as immunological, genetic and environmental [8].

The cause and cure for IBD are yet to be discovered, and therapeutic strategies are aimed towards attaining and maintaining remission from inflammatory episodes. Intestinal mucosa of patients with IBD has been previously reported and is characterized by overproduction of reactive oxygen species (ROS) and an imbalance of important antioxidants, leading to oxidative damage. Self-sustaining cycles of oxidant production may amplify inflammation and mucosal injury [7,8]. Thus, the main goal of IBD treatment is to prevent frequent recurrence of inflammation and maintain remission. Nonenzymatic therapies include drugs classified as aminosalicylate, corticosteroids, immunosuppressive agents and biological agents. Corticosteroids such as synthetic prednisone are still the most effective treatment in activated stage of IBD. These steroids act on the immune response in a wide range of ways, and patients often have long-term dependence on these drugs. 5-aminosalicylic acid (5-ASA) preparations such as mesalamine and the like are widely used for remission treatment. Chemotherapy can be administered by using azathioprine (AZA), its metabolite 6-mercaptopurine (6-MP) and methotrexate. Antibiotics such as metronidazole or ciprofloxacin are used in IBD to treat intestinal infections caused by high bacterial load (Bacteroides, Clostridium difficile). Although many patients are successfully treated with conventional medications (most relief), two-thirds require surgery. Further treatment strategies for IBD that target long-term treatment of lymphocytes and inflammatory cytokines have been designed and under investigation. Traditionally, drugs mediating these desired effects, such as salicylic acid, glucocorticoid, immunosuppressive agents, immune modulators, and other conventional drugs [9], are usually administered in high doses and/or systemically, leading to significant adverse events. Therefore, the prevention and reduction of drug-related side effects are highly challenging in IBD treatment [10].

The oral dosage form is characterized by low production cost, easy handling for the patient, accurate dose, and excellent stability and storage. In contrast, the oral route is affected by changes in intestinal absorption, metabolism of the intestinal cells, and usually the liver is encountered in the portal circulation, thereby passing the first-pass effect. In general, the oral route is the most desirable and acceptable route for administering drugs in the treatment of IBD [11]. After oral formulations are administered, the dosage forms release the active ingredients into the intestinal lumen where they are absorbed by the gastrointestinal mucosa. Finally, the active ingredient reaches the systemic circulation and is distributed throughout the body. However, systemic adverse drug reactions may also occur and may affect the quality of life of patients. The marked differences in the intestinal tract environment of the gastrointestinal tract and the differences between the healthy and inflamed intestinal regions have facilitated the development of pharmaceutical technologies that specifically deliver the active compounds to the inflamed intestinal regions. Oral drug-delivery systems for the treatment of IBD have been developed and allow more or less effective delivery of drugs to the site of disease. Based on the persistent large intestine in UC, most oral dosage forms are used to treat the colon. Therefore, it is much more difficult to treat discrete areas of inflammation in the CD by using an oral drug-delivery system [12].

**Figure 1:**
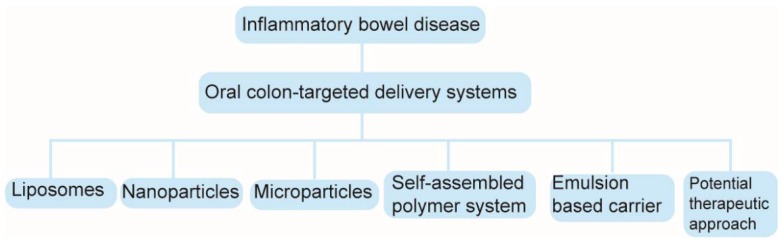
Structural diagram of oral colon-targeted delivery systems in IBD.

Targeting preparations could improve pharmacological effects and reduce adverse reactions [13]. Targeted drug delivery can be divided based on carriers. These include liposomes, microparticles, nanoparticles and emulsions. Among recent techniques used for colon-specific delivery, micro- and nanoparticles are well known for achieving site specificity and increasing drug stability via encapsulation [14]. The main objective of targeted drug strategy is to target the maximum concentration of active agents in inflamed intestinal tissues by using selective delivery to achieve therapeutic efficacy, while simultaneously reducing adverse effects. In addition, such a targeted delivery system must meet the conditions for complete biodegradation and high biocompatibility without pro-inflammatory properties [15]. Nanotherapeutics [16,17], therapeutic targets [18] and colon-targeted oral drug-delivery systems [19] for inflammatory bowel disease have been reviewed. In our review, new targeting preparations in IBD therapy are systematically reviewed. The structural diagram of these oral colon-targeted delivery systems is shown in Figure 1. This paper provides a valuable pharmaceutical strategy for studying the formula and preparation technology of oral colon-targeted delivery systems that are suitable for treating IBD.

## 2. Pharmaceutical Strategies

### 2.1. Liposomes

Liposomes are double-layer vesicle structures based on phospholipids that are enclosed in aqueous volumes. Liposomes exhibit highly compatible phospholipid vesicles that are capable of carrying hydrophilic (aqueous core) and lipophilic (in lipid bilayer) medicaments due to their amphoteric properties. A lipophilic drug is incorporated in the liposome at the same time the lipid membrane is formed, and the hydrophilic drug is dissolved in the aqueous medium. The lipid membrane is hydrated by continuous rotation, extrusion, sonication or other ways to form vesicles. These systems are designed using a controlled delivery system, but the system must be targeted and protected from changes in pH [20]. Liposomes, which are biodegradable and essentially nontoxic vehicles, can encapsulate both hydrophilic and hydrophobic materials [21]. The use of liposomes has been shown to selectively target inflamed tissues, with the disruption of the intestinal barrier function at the site of inflammation, allowing accumulation of particulate delivery carriers. Liposomes can also be modified to enhance binding and cellular uptake to diseased tissue with the use of cationic lipids or attachment of targeting ligands [22].

L. Li and his colleagues evaluated the preclinical antitumor activity of liposomal curcumin in colorectal cancer. In this study, the efficacy of liposomal curcumin with standard chemotherapeutic agents (oxaliplatin) was compared. Different ratios of total lipid to curcumin (*w/w*) were evaluated from 10:1 to 4:1. The optimized 10:1 ratio was selected based on the test to determine the optimal encapsulation efficiency of curcumin by the liposomes. In-vitro studies with curcumin liposomes and dose-dependent growth inhibition were observed, and apoptosis in LoVo and Colo205 human colorectal cancer cell lines was observed. Synergy was also observed in in-vitro LoVo cells with a 4:1 ratio of liposomal curcumin and oxaliplatin. In-vivo studies also showed significant tumor growth inhibition in Colo205 and LoVo cells, and the growth inhibition observed with liposome curcumin was higher in Colo205 cells than oxaliplatin. Antiangiogenic effects are seen when tumors from animals are treated with liposomal curcumin. By immunohistochemical observation of CD31, the expression of vascular endothelial growth factor and interleukin-8 was attenuated. Therefore, curcumin liposomes exhibit better in-vitro and in-vivo activity in colorectal cancer [23,24].

### 2.2. Nanoparticles

Granular carriers are small carrier-based lipids and polymer matrix systems, where the drug is dispersed or dissolved in a lipid or polymer matrix. These systems are called nanoparticles or microparticles, based on size. Nanoparticles (NPs) in nanoscale units consist of two types: solid lipid nanoparticles and nanostructured lipid particles. Microparticles, on the other hand, are usually micron-sized microspheres. In order to increase the efficacy of IBD treatment, these particle systems can be further modified by coating or encapsulating with alginate beads. This matrix system prevents rapid drug release and promotes controlled release by reducing mobility of the drug molecules bound to the solid matrix. The release of the drug in the matrix system is further influenced by matrix composition and particle structure [25]. NPs, with a diameter of 10–1000 nm, are drug-loaded particles that are prepared by taking natural polymers or synthetic chemicals as carriers. Drugs can be embedded or dissolved in NPs and adsorbed or coupled on their surface. Encapsulating drugs within NPs can improve the solubility and pharmacokinetics of drugs and, in some cases, enable further clinical development of new chemical entities that have stalled because of poor pharmacokinetic properties [26].

Solid lipid NPs (SLNs) are beneficial in terms of drug protection and prevention of degradation. As a result of the homogenization of SLNs, increase in entrapment efficiency and initial release results in an increase in the bioavailability of the encapsulated drug. Due to the slow degradation of the lipid matrix, SLNs have unique properties, such as micro size with high surface area, high drug-loading capacity, and extended drug release. These systems are typically prepared by applying high-pressure homogenate and ultrasonic treatment of molten lipids. Some NP carriers for the treatment of IBD are based on chitosan, poly (lactic-co-glycolic acid) (PLGA), Eudragit P-4135F, which is a new pH-sensitive polymer, and silica NPs [27]. Nanostructured lipid carriers (NLCs) are part of the nanometer linear system. These systems are mixed with solid and liquid lipids. Liquid lipids provide flexibility in the carrier system, allowing for improved drug loading. The concept behind its preparation is that the solid lipid crystals with a higher melting point form a lipid core, and then the liquid lipid forms an outer layer containing a higher amount of lipophilic drug. Advantages of this structure include providing oxidation and hydrolytic stability [28]. Table 1 shows the formula, preparation method and biological activity of different nanoparticles in treating UC.

### 2.3. Microparticles

Microparticles (MPs) used in IBD therapy often range from 1–150 µm in diameter and are designed to target inflamed intestinal tissues and/or to be internalized by immune cells. The most common methods for MP fabrication include the complex coacervation method. This involves the emulsion solvent-evaporation approach, spray-drying process, and solvent-extraction method. MPs can be divided into noncoated and coated forms. Noncoated MPs can be characterized as a system that encapsulates the drug directly into polymers [49]. Table 2 shows the formula, preparation method and biological activity of different microparticles in treating UC.

### 2.4. Self-Assembled Polymer System

Self-assembled polymer systems consist of natural and synthetic polymers, which are oriented in specific shapes or forms, or swell in the presence of water or any suitable specific polar solvent system. These systems are commonly used in antifungal and topical forms to treat ulcers and cancers.

Hydrogels are crosslinked networks of hydrophobic polymers that are physically or chemically linked to each other. The system is expanded by absorbing large amounts of water, and the drug is released by swelling or by degradation of the polymer after swelling. The intestinal mucosal region acts as a hydrogel that provides effective control of release of the drug into the inflammatory site. Hydrogels can be classified as macroporous, microporous, or nonporous according to pore size formed by the entanglement of the polymer. The pore size of macroporous hydrogels is 0.1–1 μm. The microporous water gel pore is 100–1000 Å, and the nonporous hydrogel pore is 10–100 Å [66].

In our previous study, we provided evidence for paeonol as a novel therapeutic agent in the treatment of UC, which was isolated from *Cynanchum paniculatum* (Bge.) Kitag. or *Aaeoina suffruticosa* Andr. in traditional Chinese medicine [67]. We also developed satisfactory paeonol coating tablets with pH-time-delayed controlled release in the colon [68,69]. Moreover, we prepared the colon-specific delivery system (Pae-SME-CSC) with paeonol-loaded self-microemulsion (Pae-SMEDDS), and evaluated its properties in vitro and in vivo, especially the anti-inflammatory effects on UC rats. It indicated that the developed Pae-SME-CSC was suitable for colon-specific drug delivery [70].

### 2.5. Emulsion-Based Carrier

These carriers are formed by the dispersion of two or more immiscible liquids stabilized by a surfactant or an emulsifier. The emulsifier causes the liquid to disperse evenly into the continuous liquid medium and produce a physical exclusion between the droplets to avoid coalescence by coating the droplets and lowering the interfacial tension [71].

Microemulsions are thermodynamically stable isotropic dispersions. The biphasic immiscible liquid is stabilized by the interfacial membrane of the surfactant molecule bound to the co-surfactant. The relative concentration of these three components can be estimated by constructing a ternary phase diagram. The components are oil in water (*o/w*) or water in oil (*w/o*), in the range of 5–100 nm. Microemulsions (*o/w* and *w/o*) improve the oral bioavailability of drugs. These have additional formulation advantages, thermodynamic stability, easy sterilization by filtration, small droplet size, and high surface area, which provide increased surface area for absorption and delivery of drug molecules. Release from the microemulsion is controlled by the interaction between the drug surfactant and the distribution of the drug between the oil phase and the aqueous phase. These systems can be highly developed for the treatment of internal inflammatory diseases [72].

Nanoemulsions are a uniform population of particle droplets consisting of long-term thermodynamically stable lipids and surfactants. These droplets are usually composed of lipid monolayer surrounding the liquid lipid core. Nanoemulsions are prepared by high-pressure homogenization, which results in the formation of droplets of uniform size. Using nanoemulsions as a carrier system has already been studied for broad-spectrum antimicrobial activity against microorganisms. In-vivo studies reveal its efficacy for treating vaginal, fungal and respiratory infections against the skin and mucous membranes. Moreover, this may be effective in treating gastritis [73].

### 2.6. Potential Therapeutic Approach

The complement system is widely considered to protect the host from invading microorganisms. However, previous studies examining the activation of the complement system have shown that it may play a detrimental role in the pathogenesis of many inflammatory and immune diseases. Complement activation products include complement components (C) 3a, C4a, C5a and C5b-9, and membrane-attack complexes. The complement activation product complement component 5a (C5a) is a potent inflammatory peptide with a broad spectrum of functions. In-vivo and in-vitro studies have demonstrated that C5a plays an important role in inflammation. Li Zhiping studied the role of C5a in IBD using an experimental mouse colitis model. Colitis was induced in mice using 2,4,6-trinitrobenzene sulphonic acid (TNBS), followed by administration of C5a aptamer by intraperitoneal injection. The clinical signs of the disease, the histopathological analysis of the colon and the level of inflammatory components were examined. Symptoms of colitis, including altered behavior, weight loss, colon damage and increased inflammatory cytokines, attenuated after treatment of mice with TNBS-induced colitis containing C5a aptamers. By phenotypic observation, histological examination and levels of inflammatory cytokines demonstrated that aptamer-treated mice exhibited significant colitis-attenuating effects compared to untreated mice. Colitis is characterized by an imbalance between proinflammatory and anti-inflammatory media. Current research results show that C5a may play a key role in IBD inflammation [74].

## 3. Pharmacokinetic Studies

Pharmacokinetics is used to evaluate pharmaceutical preparations for slow-release drug-delivery systems. First, studying pharmacokinetic properties allows us to understand the absorption, distribution, metabolism and excretion characteristics of drugs in the body. Related pharmacokinetic parameters, combined with the physical and chemical properties of drugs, pharmacodynamic properties and clinical needs, help determine drug preparation’s necessity [75]. During drug preparation into a sustained-release drug-delivery system, pharmacokinetic principles are used to design dosage form, dose, release mode, release time, and other factors. In addition, pharmacokinetic studies are used to evaluate and monitor whether the system achieves the desired effect of sustained drug release. In recent years, the development of modern instrumental analysis technology brought new technologies for pharmacokinetic studies. These include liquid chromatography, ion-selective electrode method, gas chromatography, mass spectrometry, and application of tandem mass spectrometry in the detection of drug concentration in chemical drugs by modern instrumental analysis methods [76].

Ye Liu applied HPLC analysis using a Dikma Diamonsil C18 on a Shimadzu LC-20A HPLC system with an ultraviolet detector at room temperature. The wavelength of the ultraviolet detector was 245 nm. Water and ethanol (57:43 *v/v*) were used as the mobile phase at a flow rate of 1 mL/min. The prolongation of the half-life (t_1/2_), enhanced residence time (mean residence time, MRT), and decreased total clearance (CL) indicated that BUD microspheres could prolong the acting time of BUD in vivo [60]. Srinivas Mutalik prepared novel pH-sensitive hydrolyzed polyacrylamide-grafted xanthan gum (PAAm-g-XG) nanoparticles (NPs) loaded with curcumin for colonic delivery. Curcumin was better absorbed systemically in nanoparticulate form with increased *C_max_* (3-fold) and AUC (2.5-fold) than when delivered as free curcumin [43]. Hiraku Onishi prepared and evaluated simple Eudragit S100 microparticles loaded with prednisolone (ES-MP) and Eudragit S100-coated chitosan-succinyl-prednisolone conjugate microparticles (Ch-MP/ES) in vitro. It was demonstrated that Ch-MP/ES could enhance the efficacy of PD and reduce the toxic side-effect of PD, while ES-MP could hardly improve the effects of PD. Only Ch-MP/ES significantly changed in-vitro and in-vivo characteristics and was found to improve PD’s in-vivo function [77].

## 4. Conclusions

IBD is a chronic disease that has an immunization period when the disease is not active. Hence, most patients need to maintain drug therapy to relieve symptoms and shorten the number and severity of seizures [78]. Treatment of IBD is minimal, but some drugs can reduce the severity of inflammation, increase the duration of remission, and reduce the risk of more serious health problems, such as colorectal cancer. Years of research have demonstrated the suitability of the colon as an absorption site, especially in GI diseases [79]. Conventional drug-delivery systems rely primarily on several nonstable parameters in the gastrointestinal tract, such as changes in pH and local enzyme-induced drug release. New drug-delivery systems, particularly multiparticle systems such as microspheres and nanoparticles, exhibit higher drug-delivery capability due to their specific accumulation, spread and long-term retention in the target inflammatory tissue compared with conventional single-unit modes [80]. Unfortunately, the problem associated with the nanoparticle process is it exhibits altered physical and chemical properties and potential risk of causing possible toxicity compared with its larger counterpart. Abiotic nanoparticle carriers can alter normal cellular activity and cause cytotoxicity because the particles cause wrinkling to the cell membrane, cytoskeleton rearrangement, and phagocytosis leading to their entry into phagocytic cells [81]. Thus, biotechnology systems with fewer side effects may have great potential in future IBD treatments. A highly biological approach in IBD treatment with a drug-delivery system does not alter normal cellular function. This may be the best method to achieve satisfactory effects in IBD therapy. Although studies on the effects of new multiparticle systems in the human gastrointestinal tract during IBD treatment are limited and need further exploration, we see that these systems will be used in combination with new biological agents in the near future to achieve maximum targeted drug efficacy at lower drug doses and side effects due to their superior advantages, such as sustainability and controlled release. 

## Figures and Tables

**Table 1:** Studies on the formula, preparation method and biological activity of different nanoparticles in treating ulcerative colitis (UC).

Categories	Carrier Materials	Pharmaceutical Ingredients	Preparation Methods	Biological Activity	Ref.
Nanoparticles	Polymethacrylate (Eudragit RL)	Clodronate	Modified solvent-displacement method	Confirmed therapeutic benefit of ClNP in vivo	[29]
Nanoparticles	Poly (lactic acid) poly (ethylene glycol) block copolymer (PLA-PEG)	TNFα siRNA	Double emulsion/solvent evaporation	Powerful and efficient nanosized tools for delivering siRNAs into colonic macrophages	[30]
Nanoparticles	---	Lipids, proteins, microRNAs (miRNAs), and ginger bioactive constituents (6-gingerol and 6-shogaol)	Derived from edible ginger	Improve inflammatory bowel disease (IBD) prevention and treatment with an added benefit of overcoming limitations such as potential toxicity and limited production scale	[31]
Nanoparticles	Polymeric mixtures of poly (lactic-co-glycolic) acid (PLGA)	Budesonide	Oil/water (*O/W*) emulsion-evaporation technique	An efficient delivery system for targeted drug delivery to the inflamed intestinal mucosa	[32]
Nanoparticles	PLGA 50:50	Betamethasone	Oil-in-water solvent-evaporation method (simple oil/water emulsification technique)	Stable targeting moiety in the gastrointestinal tract	[33]
Nanoparticles	Eudragit FS30D, Eudragit RS100	Budesonide	Oil-in-water emulsion method	An effective oral colon-targeted delivery system for colitis therapy	[34]
Nanocapsules	Eudragit S100	Prednisolone	Nanoprecipitation method	Provide effective way of treatment of colonic disease	[35]
Nanoparticles	Polymeric mixtures of poly PLGA and a pH-sensitive methacrylate copolymer	Budesonide	An adaption of the modified spontaneous emulsification solvent diffusion method	Useful for colon-specific delivery in inflammatory bowel disease	[36]
Nanoparticles	PLGA	Budesonide	Oil-in-water (*O/W*) emulsion technique	Targeted drug delivery to the inflamed intestinal mucosa	[37]
Nanostructure lipid carriers (NLCs)	Precirol ATO^®^5, Miglyol 812	Budesonide	High-pressure homogenization	A targeted drug-delivery system for IBD treatment	[38]
Nanoparticles	Trimethylchitosan (TMC) Eudragit^®^ S100 PLGA, PEG-PLGA and PEG-PCL	Ovalbumin (OVA)	Water-in-oil-in-water solvent-evaporation method, ionic complexation/gelation method	The highest accumulation of ovalbumin (OVA) in inflamed colon	[10]
Nanoparticles	PLA	CD98 Fab′-bearing quantum dots (QDs)	A modified oil-in-water (*O/W*) emulsion solvent-evaporation technique	Active colitis-targeted delivery	[39]
Nanoparticles	EC	Betamethasone	Emulsification solvent-evaporation technique	A significantly higher mitigating effect	[40]
Nanoparticles	Eudragit RL PO	Silybin	Solvent-evaporation emulsification technique	Reduced TNF-a, IL-6 and MPO activity significantly	[41]
Nanoparticles	Enzyme-sensitive azo-polyurethane and pH-sensitive methacrylate copolymer	Budesonide	A quasiemulsion solvent diffusion with some modifcations	An effective and safe colon-targeted delivery system for colitis therapy	[42]
Nanoparticles	Novel pH-sensitive hydrolyzed polyacrylamide-grafted xanthan gum (PAAm-g-XG)	Curcumin	A modified version of the solvent-evaporation cross-linking technique	Suitable for colon targeting	[43]
Silica nanoparticles (SiNPs)	Silica	5-Amino salicylic acid (5ASA)	---	Combine advantages from selective drug targeting and prodrugs	[44]
Nanoparticles	Eudragit S100 (EU S100)	5-Aminosalicylic acid (5-ASA)	Supercritical fluids (SEDS) technique	5-ASA was imbedded into EU S100 in an amorphous state after SEDS processing and the SEDS process did not induce degradation of 5-ASA	[45]
Nanoparticles	Oxidation-responsive b-cyclodextrin material (OxbCD)	Tempol (Tpl)	A modified nanoprecipitation/self-assembly method	Reduce ulcerative colitis in mice effectively	[46]
Nanoparticles	EudragitR S100	Curcumin–celecoxib combination	Emulsion solvent-evaporation technique	More efficacious than nanoparticles of either drugs or drug suspension	[47]
Nanovesicles	Hydrogenated soy phosphatidylcholine-coating polyethylene glycol-containing vesicles with chitosan and nutriose	Quercetin	---	A marked amelioration of symptoms of 2,4,6-trinitrobenzenesulfonic acid-induced colitis	[48]

**Table 2:** Studies on the formula, preparation method and biological activity of different microparticles in treating UC.

Categories	Carrier Materials	Loaded-Ingredients	Preparation Methods	Biological Activity	Ref.
Microsphere	Chitosan-alginate	Icariin	Emulsification-internal gelation technique	Exert the colon-protective effects through reducing the inflammatory response	[50]
Microsphere	Eudragit S100 liquid paraffin	Metronidazole	Emulsification solvent-evaporation method	Enhance drug entrapment, and effect the drug release	[51]
Microsphere	PLGA microsphere	Glucagon-like peptide-2	Solid-in-oil-in-water (*S/O/W*) method	Resistant to degradation and decreased the severity of dextran sulfate sodium (DSS)-induced ulcerative colitis	[52]
Microspheric vehicle	Microspheric vehicle formed by cationic konjac glucomannan (cKGM), phytagel	An antisense oligonucleotide against TNF-α	Water-in-oil (*W/O*) emulsion method	Significantly decreased the local level of TNF-α and alleviated the symptoms of colitis in the mice	[53]
Microsphere	pH-triggered Eudragit-coated chitosan microspheres	Curcumin	Emulsion crosslinking method followed by coating with Eudragit S-100	A promising system for pH-dependent delivery of drug to colon in ulcerative colitis	[54]
Microsphere	The enzyme diamine oxidase (DAO) in CaCMS/alginate microspheres	The enzyme diamine oxidase (DAO)	---	A procedure able to afford protection of the entrapped enzyme against gastrointestinal degradation	[55]
Microsphere	Colon-targeted microspheres which were compressed into tablets using the enzyme-dependent polymer (pectin) as coat	The nonsteroidal anti-inflammatory bumadizone calcium dihydrate	Quasi-emulsion solvent-diffusion method	Achieved significant decrease in myeloperoxidase activity and inflammation with delayed T_max_ (4 h) and lower C_max_ (2700 ng/mL) when compared to marketed product	[56]
Microsphere	Hydrogel microspheres of chitosan grafted with vinyl polymers	5-Aminosalicylic acid (5-ASA)	Water-in-oil (*W/O*) emulsification method	Exhibited better therapeutic effects in comparison to 5ASA plain drug solution in oral administration	[57]
Microsphere	Chitosan microspheres	5-ASA and camylofine dihydrochloride	Emulsion method followed by enteric coating with Eudragit^®^ S-100	Specific delivery of drug to the colon and reduce symptoms of ulcerative colitis	[58]
Microsphere	Eudragit L100 (EuL)-coated chitosan (Ch)–succinyl-prednisolone (SP) conjugate microspheres (Ch SP-MS/EuL)	Prednisolone (PD)	---	Enhanced effectiveness of PD and reduced toxic side effects of PD greatly	[59]
Microsphere	Budesonide (BUD) guar gum microspheres	Budesonide (BUD)	Emulsion crosslinking technique	Prolong the acting time of BUD in vivo	[60]
Microsphere	Chitosan microparticles	Mesalamine	Emulsion chemical crosslinking technique	Maintain the drug concentration within target ranges for a long period of time	[61]
Microparticle	Kafrin microparticles	Prednisolone	A phase-separation method	The majority of the loaded prednisolone was not released in in-vitro conditions simulating the upper gastrointestinal tract	[62]
Microparticle	*N*-Succinyl-chitosan (SucCH) microparticle	5-ASA	Spray-drying method	Improved efficacy in the healing of induced colitis in rats	[63]
Microsphere	pH-sensitive microspheres using Eudragit P4135F	Low-molecular-weight heparins (LMWH)	A double emulsion technique with either solvent extraction or evaporation	Exhibited a particle size adapted to the needs of inflammatory bowel disease therapy, an efficient LMWH encapsulation, and a pH-controlled drug release	[64]
Microparticle	Poly-ε-caprolactone (PCL) celecoxib-loaded microparticles	Celecoxib	Solvent-diffusion technique	Enhanced the bioavailability and extended the duration of drug-plasma concentration in rats	[65]
